# Panoramic volumetric clinical handheld photoacoustic and ultrasound imaging

**DOI:** 10.1016/j.pacs.2023.100512

**Published:** 2023-05-18

**Authors:** Changyeop Lee, Seonghee Cho, Donghyun Lee, Jonghun Lee, Jong-Il Park, Hong-Ju Kim, Sae Hyun Park, Wonseok Choi, Ung Kim, Chulhong Kim

**Affiliations:** a Departments of Electrical Engineering, Convergence IT Engineering, Mechanical Engineering, and Medical Science and Engineering, Medical Device Innovation Center, Pohang University of Science and Technology, Pohang 37673, Republic of Korea; b Division of Cardiology, Department of Internal Medicine, Yeungnam University Medical Center, Yeungnam University College of Medicine, Daegu 42415, Republic of Korea; c Division of Cardiology, Department of Internal Medicine, Severance Hospital, Seoul 03722, Republic of Korea; d Division of Cardiology, Department of Internal Medicine, Daegu Veterans Hospital, Daegu 42835, Republic of Korea; e Department of Biomedical Engineering, College of Medicine, The Catholic University of Korea, Seoul 06591, Republic of Korea

**Keywords:** Handheld system, Photoacoustic/ultrasound imaging, Three-dimensional imaging, Panoramic imaging, Clinical translation

## Abstract

Photoacoustic (PA) imaging has gained much attention, providing structural and functional information in combination with clinical ultrasound (US) imaging systems. 2D PA and US imaging is easily implemented, but its heavy dependence on operator skills makes 3D imaging preferable. In this study, we propose a panoramic volumetric clinical PA and US imaging system equipping a handheld imaging scanner weighing 600 g and measuring 70 × 62 × 110 mm^3^. Multiple PA/US scans were performed to cover a large field-of-view (FOV), and the acquired PA/US volumes were mosaic-stitched after manually correcting the positions and rotations in a total of 6 degrees of freedom. PA and US maximum amplitude projection images were visualized online, while spectral unmixed data was quantified offline. The performance of the system was tested via tissue-mimicking phantom experiments. The system’s potential was confirmed in vivo by panoramically imaging vascular networks in human arms and necks, with FOVs of 331 × 38 and 129 × 120 mm^2^, respectively. Further, we quantified hemoglobin oxygen saturation levels in the radial artery, brachial artery, carotid artery, and jugular vein. We hope that this system can be applied for various clinical fields such as cardiovascular imaging, dermatology, vascular surgery, internal medicine, and oncology.

## 1 Introduction

Photoacoustic (PA) imaging has become a well-known non-invasive in vivo imaging modality with great potential for widespread clinical use. When optical absorbers in tissues are excited with pulse lasers, the resulting PA waves can be received by ultrasound (US) transducers and processed into PA images [1], [2], [3]. Endogenous contrast absorbers (e.g., melanin, lipids, water, oxy-hemoglobin (HbO_2_), and deoxy-hemoglobin (Hb)) possess different light absorption spectra [4], and thus PA imaging can selectively highlight each component according to its laser wavelength. In this way, PA imaging can beneficially provide such clinical information as vessel networks, vessel density, hemoglobin oxygen saturation (sO_2_), and chromophores’ concentrations [5], [6]. This information is variously useful in diagnosing cancerous, hemorrhagic, ischemic, cardiovascular, and skin diseases. Compared to such conventional medical imaging techniques such as computed tomography (CT), magnetic resonance imaging (MRI), and positron emission tomography (PET), PA imaging is free from ionizing radiation, does not require external contrast agents for angiographic imaging, and is relatively compact and affordable [7], [8], [9], [10], [11], [12].

PA imaging is readily paired with clinical US imaging platforms to form a bi-modal PA/US imaging modality that provides rich functional and structural information [13]. Cross-sectional PA and US images are easily formed in 2D space, but the image results depend heavily on the operators’ skill, and the lack of reproducibility can lead to inaccurate diagnoses. On the other hand, 3D imaging can minimize operator dependency by providing relatively accurate structures and functions within a region of interest (ROI) [14]. Various 3D PA/US imaging techniques have been developed using different types of custom and commercial US transducers [5], [15], [16], [17], [18], [19], [20], [21], [22], [23], [24], [25], [26], [27], [28].

3D PA imaging systems have been developed with a custom-made 2D spherical array, or alternatively with 1D circular and arc array US transducers with motorized stages [15], [16], [19], [20], [21], [22], [23], [29], [30]. Both approaches are preferred for PA imaging because PA waves propagate in a spherical shape from the wave origin. However, they are implemented on customized transducers which are relatively expensive, and require complex systems and high computational resources. Further, these specialized US transducers are not appropriate for US imaging due to large inter-element gap (i.e., pitch) and thus not routinely used in clinical practice. Conventional handheld matrix array US transducers can be another way to provide 3D imaging, but their small apertures provide low resolution and are hampered by the limited view effect [31]. Similar to custom-optimized US transducers for PA imaging, matrix array US transducers also require expensive and complicated electronics to cover a large number of transducer elements (e.g., 32 × 32) [18], [32]. Conventional 1D array US transducers with motorized stages can perform 3D imaging by translating the transducer in the elevation direction and stacking the 2D images. This approach is relatively simple and cost-effective to implement, and friendlier for human studies because it is usually based on clinically-viable US machines. However, 3D imaging systems using these 1D array US transducers combined with linear stages are usually heavy and bulky, making handheld operation difficult [17], [24], [25], [26], [28].

To overcome the limitation, we previously developed a custom three-dimensional clinical handheld PA/US imaging scanner using the scotch yoke mechanism that enables the use of a small and light weight motor instead of a linear stage [27]. The scanner weighed 950 g, had a field of view (FOV) of 38.4 × 40 mm^2^, and dimensions of 100 × 80 × 100 mm^3^, allowing for handheld operation. However, the scanner’s size and weight were not sufficiently small to image weight-sensitive and small areas, such as the human neck, but minimizing the size would unwantedly reduce the FOV. The acoustic coupling was provided using a water tank sealed with polyvinyl chloride (PVC) membrane, which formed multiple layers of acoustic impedance mismatch that could hamper the signal-to-noise ratios (SNRs) for deep tissue imaging. Further, previewing of the 3D images (e.g., maximum amplitude projection (MAP)) was only available in post-processing, and thus we could not confirm the scanning results during the imaging experiments [33].

In this study, we present a newly updated three-dimensional clinical handheld PA/US imaging scanner weighing 600 g, measuring 70 × 62 × 110 mm^3^ that provides both a FOV of 38.4 × 25 mm^2^ and a three-dimensional PA/US imaging. The scanner uses a transparent solid US gel pad that effectively acoustic impedance-matching, enabling improved SNRs. To overcome the limitation of the reduced FOV of the updated 3D imaging scanner, we demonstrated panoramic imaging where multiple volume images are mosaic-stitched after correcting the volume position and rotation in a total of 6 degrees of freedom (DOFs). In addition, we implemented online PA MAP display to preview the panoramic scan results on-site. We performed tissue-mimicking phantom experiments to confirm that the SNRs improved on average by 10.8 dB compared to the previous system while maintaining image resolutions. Further, we conducted super-wide-FOV panoramic in vivo imaging experiments on a human arm and neck, where the FOVs of the arm and neck images were 331 × 38 mm^2^ and 129 × 120 mm^2^, respectively. The maximum PA penetration depth in the volunteer human subject was at least 28.6 mm, an increase of 13.9 mm compared to the previous system. In addition, PA sO_2_ values were measured in the radial artery (RA, 95 ± 7.3%), brachial artery (BrA, 97 ± 6.9%), carotid artery (CA, 97 ± 8.2%), and jugular vein (JV, 84 ± 12.8%) [34,35]. The obtained results suggest that the updated 3D clinical PA/US imaging scanner and panoramic imaging modality can become practical imaging tools for a variety of clinical uses including cardiovascular, skin, vascular surgery, internal medicine, and tumor diagnosis.

## 2 Materials and methods

### 2.1 3D clinical PA/US imaging system

**Fig. 1:**
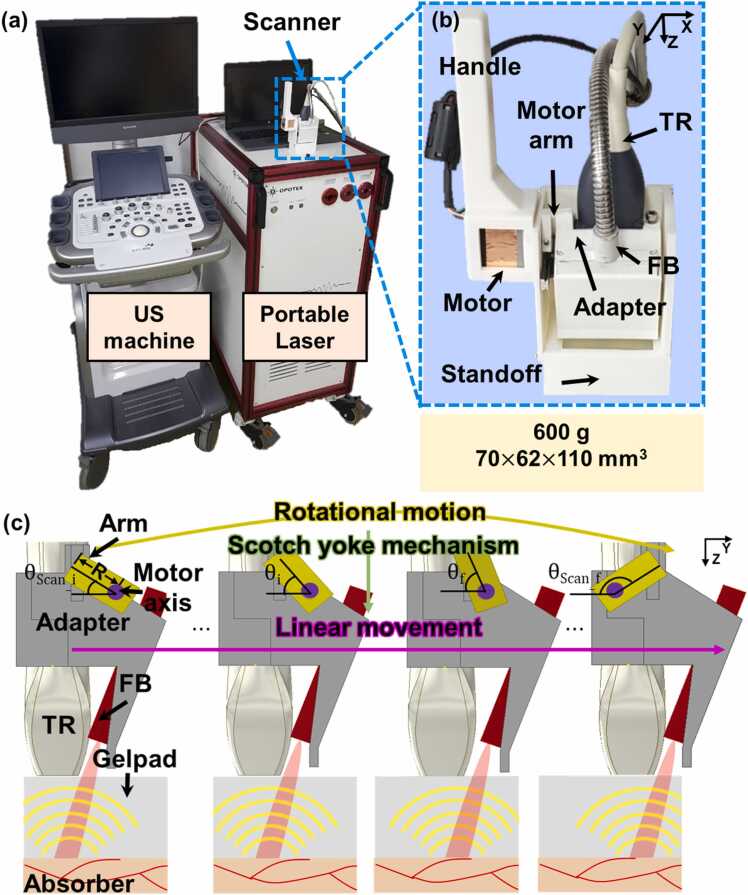
Photographs of (a) a 3D clinical PA/US imaging system and (b) handheld imaging scanner. (c) Schematic of the 3D handheld scanning managed by the scotch yoke mechanism. FB, fiber bundles; PA, photoacoustic; TR, an ultrasound transducer; US, ultrasound; $\theta_{\mathrm{Scan\_i}}$ and $\theta_{\mathrm{Scan\_f}}$, scan angles of first and last imaging positions, respectively; $\theta_{i}$ and $\theta_{f}$, scan angles of previous and subsequent imaging positions, respectively; and R, the length of the motor arm.

Fig. 1 shows the newly updated 3D clinical PA/US imaging system and the handheld scanner. The system consisted of an 128-element linear array US transducer (L3–12, Alpinion Medical Systems, Republic of Korea) with a center frequency of 8.5 MHz, a fractional bandwidth of ≥ 62% at − 6 dB, and average and standard deviation of the peak to peak sensitivity of − 50.53 and < 1 dB, respectively, a US imaging machine (EC-12R, Alpinion Systems, Republic of Korea) with 64 data acquisition channels and a sampling frequency of 40 MHz, a portable optical parametric oscillator (OPO) laser system (Phocus Mobile, OPOTEK, USA) with the maximum pulse repetition frequency (PRF) of 10 Hz, and the updated 3D handheld scanner (Fig. 1a). PA imaging was implemented at 5 Hz in maximum because the US machine had 64 data acquisition channels and thus two laser pulses were used to acquire full-aperture PA data from a 128-element transducer (Supplementary Fig. S2). The updated scanner consisted of a handle, a motor arm, an adapter, a US transducer, a transparent US gel pad (WATER GEL, Blue Ocean Medical Technology, Republic of Korea) that works as a standoff (Fig. 1b), a linear fiber bundle (TFO-VIS100SL46–2000-F, Taihan Fiberoptics, Republic of Korea), and a step motor (PKP523N12A, Ina Oriental Motor, Japan). The US gel pad had a frequency-to-acoustic attenuation coefficient similar to that of water and did not contain any PVC membrane, which provided better sound transmission efficiency than the standoff in the previously designed scanner [36]. The axial dimension of the gel pad was fabricated to be 30 mm to avoid PA reflection artifacts up to 30 mm depth from the skin [33]. The handle, motor arm, probe adapter, housing, and standoff holder were all made with a 3D printer (i3 MEGA, Anycubic, China), using white filament to minimize light absorption. In order to prevent distortion or torque that may occur when driving on one side, a groove that serves as a guide rail was included on the housing on the opposite side of the drive unit. The scanner was measured to be 70 × 62 × 110 mm^3^ and weighed 600 g, values that were respectively 40% and 37% less than those of the previously designed scanner [27]. The 3D handheld scanner was managed by the scotch yoke mechanism that converted rotational motion to linear reciprocating movement (Fig. 1c). While imaging and data acquisition were conducted, the step motor began to rotate the arm to generate torque at the arm tip which is connected to the vertical groove of the probe adapter. The horizontal component of the force moved the adapter linearly in the scanning direction while the vertical component did not affect the motion because the arm tip moved freely along the groove. The detailed description for the scotch yoke mechanism is explained in Supplementary Fig. S1 and the operation of the mechanism could be found in the **Supplementary Video S1** in [27]. The new scanner provided a full scan range of 25 mm along the y axis (i.e., the elevation direction) and the lateral width of the transducer was 38.4 mm, and thus the FOV for one 3D PA/US image was 38.4 × 25 mm^2^ along the x (i.e., the lateral direction) and y-axes, respectively.

Supplementary material related to this article can be found online at doi:10.1016/j.pacs.2023.100512.

The following is the Supplementary material related to this article Video S1..

Video S1

### 2.2 Data acquisition and scanning process

**Fig. 2:**
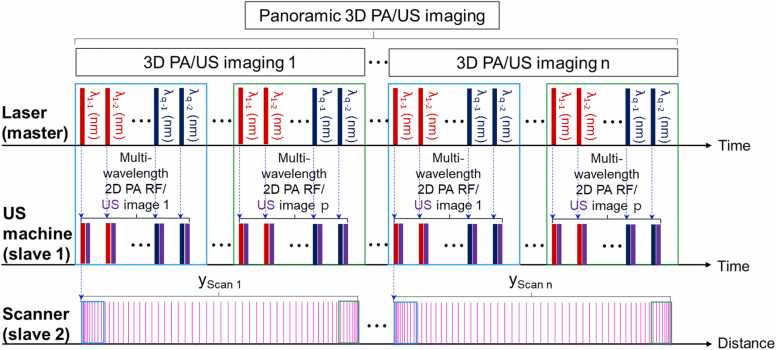
A timing chart for 3D PA/US data acquisition and scanning. PA, photoacoustic; US, ultrasound; $y_{\mathrm{Scan}}$, the scan range for one 3D scan; p, the number of multi-wavelength 2D PA RF/US image; q, the number of wavelengths used; and n, the number of the 3D imaging for the panoramic 3D imaging.

Fig. 2 shows a timing chart of the 3D PA/US data acquisition and scanning process. When the US imaging machine (i.e., slave 1) and scanner system (i.e., slave 2) received triggers from the laser system (i.e., master), PA/US image data was acquired and scanning was performed simultaneously. 3D scanning was governed by the scotch yoke mechanism, and thus, the imaging step sizes, indicated by pink columns, changed sinusoidally while the scanning was being performed. The jade and green boxes on pink columns indicated example imaging step sizes, and the imaging step size, dy, is expressed as an equation using R, $\theta_{i}$, and $\theta_{f}$ (Fig. 1c):

(1) $$\mathrm{dy}=R\times (\cos\theta_{i}-\cos\theta_{f}),$$

where R is the length of the motor arm, and $\theta_{i}$ and $\theta_{f}\;$are the scan angles of the previous and subsequent imaging positions, respectively. The dy was set to avoid spatial aliasing in the scan direction and ranges from 0.19 to 0.35 mm, which is less than half the US transducer’s elevation beam-width (∼ 1 mm at −6 dB round-trip intensity, at 30 mm imaging depth). The maximum scan range for one 3D scanning, $y_{\mathrm{Scan}}$ is expressed in the same way:

(2) $$y_{\mathrm{Scan}}=R\times (\cos\theta_{\mathrm{Scan\_i}}-\cos\theta_{\mathrm{Scan\_f}}),$$

where $\theta_{\mathrm{Scan\_i}}$ and $\theta_{\mathrm{Scan\_f}}$ are the scan angles of the first and last imaging positions (Fig. 1c). Because the R, $\theta_{\mathrm{Scan\_i}}$, and $\theta_{\mathrm{Scan\_f}}$ are 15 mm, 32°, and 145°, respectively, the $y_{\mathrm{Scan}}$ was calculated as 25 mm. Thus, the FOV for one full scan, ${\mathrm{FOV}}_{\mathrm{scan}}$, is equal to 38.4 × 25 mm^2^. The scan time for one 3D scanning, $T_{\mathrm{Scan}}$, is represented as follows:

(3) $$T_{\mathrm{Scan}}=T_{\mathrm{Image}}\times p\times q=16.6\times \mathrm{q sec}$$

where $T_{\mathrm{Image}}$ is the PA/US image acquisition period (0.2 s), p is the number of 2D PA/US images in one full scan, and q is the number of wavelengths. For p, we used a constant number of 83 to make dy small enough to avoid spatial aliasing in the scanning direction. To ensure same dy regardless of the number of wavelengths (i.e., q), the step motor rotated q times slower and thus the $T_{\mathrm{Scan}}$ was multiplied by q. As soon as one full scan was finished, PA/US imaging was freezed and the arm of the scanner moved counterclockwise to return the probe to the initial position to prepare for the next scan. For each 3D imaging, the scan position was adjusted manually for panoramic 3D imaging. The scan time, range, and FOV for the panoramic imaging increased according to the number of 3D PA/US imaging rounds (i.e., n) to cover the desired ROI.

### 2.3 Online and offline panoramic 3D PA/US signal and image processing

**Fig. 3:**
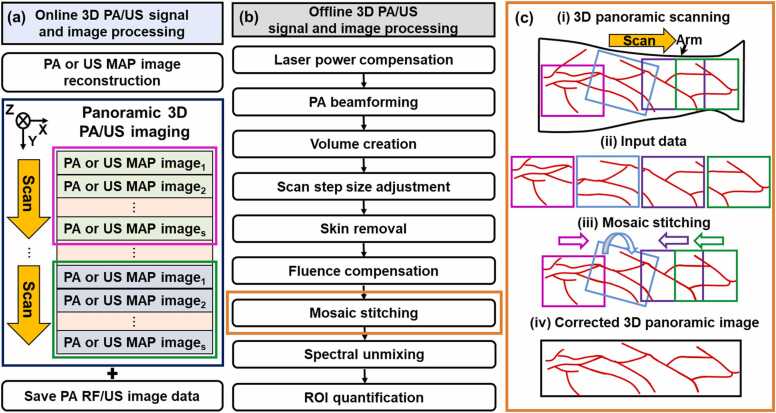
Diagrams of (a) online and (b) offline 3D PA/US signal and image processing. (c) Detailed schematic of the mosaic stitching process. PA, photoacoustic; US, ultrasound; p, the number of single-wavelength MAP imaging for one 3D PA/US imaging; MAP, maximum amplitude projection; ROI, region of interest; s, the number of single-wavelength 2D PA RF/US image; q, the number of wavelengths used; and n, the number of the 3D imaging for the panoramic 3D imaging.

Fig. 3 schematically shows the 3D PA/US signal and image processing steps in online (i.e., on the US machine) and offline (i.e., on a separate personal computer). During the scanning, cross-section PA or B-mode US images were reconstructed and visualized online in real-time using a variety of beamforming methods (e.g., delay-and-sum (DAS), delay-multiply-and-sum (DMAS), and Fourier reconstruction) [33]. At the same time, PA MAP or US maximum intensity projection (MIP) images were displayed and cumulatively updated as soon as each cross-section PA/US image was acquired. A single wavelength data was selected among multiple wavelength PA data to be displayed as the representative preview image for online PA MAP imaging. While image reconstruction was being conducted, 2D PA radio-frequency (RF) data and US image data were saved sequentially on the US machine (Fig. 3a). When the data acquisition was completed, the data were post-processed on the US machine or transferred via a storage device to a PC for offline processing. The pulse energy of every laser pulse output was recorded by the energy meter. To calibrate the pulse energy variation, PA RF data was divided by the corresponding laser pulse energy before PA image reconstruction via Fourier domain reconstruction in the k-Wave MATLAB toolbox [37]. Due to the narrow elevation beam-width of the US transducer, elevation beamforming was not performed [38,39]. 2D PA/US image data were stacked along the elevation scan direction to form PA/US volume data. Because the scotch yoke mechanism created sinusoidal step sizes, the PA/US volume data were interpolated in the scan direction to make the step size uniform (0.059 mm) [27]. Skin removal was applied to the PA images by detecting the skin contour from the US image and segmenting each cross-sectional PA image from a depth of 0.77 mm (i.e., 40 pixels) from the skin contour. The segmented PA images were then used to compensate for light fluence variations by normalizing each cross-sectional PA image by the background PA signal for each depth and wavelength [40]. To correct arbitrary volume positions and merge the volumes in a common coordinate (Fig. 3ci-ii), manual mosaic stitching (Fig. 3ciii-iv) was performed using the 3D Photoacoustic Visualization Studio (3D PHOVIS) [41], [42], [43]. While the previous version of 3D PHOVIS allowed only the linear translation of the volumes, we updated the software to allow rotation of 3D images for flexible stitching in a total of 6 DOFs. Based on this new function, manual stitching was performed by translating or rotating 3D images to appropriate positions in all directions based on the overlapping patterns in US structure and PA vascular images (Fig. 3c-iii). Because biological soft tissues were deformable and the laser illumination was not uniform, volume stitching could create seams or duplicate artifacts in the overlapping regions. To solve the problem, we applied various apodization (i.e., window weighting) options on the overlapping regions of PA/US volumes. After position correction, automatic zero-padding was performed on each volume so that each volume matched the overall size of the panoramic volume (Fig. 3c-iv). Linear spectral unmixing was performed to unmix PA HbO_2_ and PA Hb values using multi-wavelength PA volumes acquired at 756 nm (Hb-dominant), 797 nm (isosbestic point), and 866 nm (HbO_2_-dominant). Spectral-unmixed PA HbO_2_ and PA Hb values less than zero (due to overfitting) were truncated to zero to eliminate false calculations of PA sO_2_. While it is desirable to use more wavelengths for accurate spectral unmixing, we minimized the number of wavelengths to make the scanning time tolerable for clinical situations [27,44]. To reduce erroneous peak values in the spectrally unmixed images caused by imperfect pixel positions in different wavelengths, we applied smoothing filters (e.g., median filter) before and after spectral unmixing. Pixel indices corresponding to the maximum PA HbO_2_ concentration in the axial direction were used to project PA sO_2_ values to create PA sO_2_ MAP images. For quantification, ROI was manually drawn in each US frame to form a volumetric ROI, and the mean and standard deviation of all PA sO_2_ values in the ROI were calculated. All offline processing was performed using MATLAB R2021b, MathWorks, USA.

## 3 Results and discussion

### 3.1 Performance benchmark via phantom experiments

**Fig. 4:**
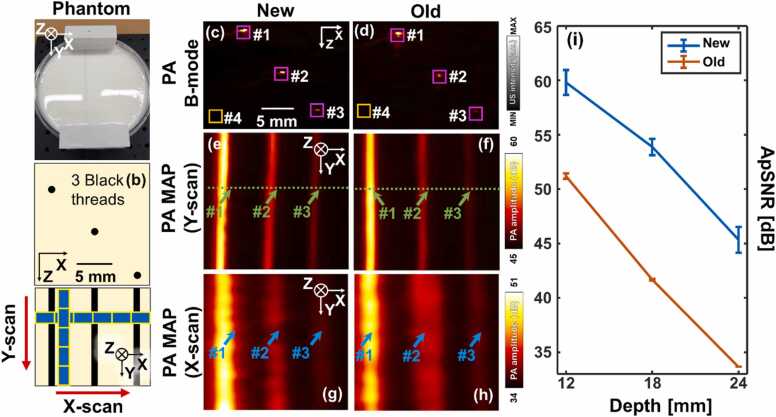
Performance comparison of systems via phantom experiments. (a) A photograph of tissue-mimicking phantom. (b) Schematics of the cross-sections of light-absorbing materials in the phantom and description of X and Y-scans. B-mode PA images acquired with (c) the new system and (d) a previously designed handheld imaging system [25]. #1-#3 represent locations of threads at different depths. Pink boxes indicate signal areas, and the #4 and gold boxes indicate noise areas selected for the quantifying pSNR. The phantom’s PA MAP images acquired with (e) the new and (f) the previously designed imaging systems. The phantom’s PA MAP images obtained with (g) the newly designed and (h) the previously designed imaging systems. (i) A graph that shows quantified ApSNRs of the threads in the whole volume data sets acquired with the newly updated and previously designed handheld scanners. pSNR, peak signal-to-noise ratio; PA, photoacoustic; ApSNR, average peak signal-to-noise ratio; and MAP, maximum amplitude projection.

The system performances of the newly updated and the previous imaging systems were compared in a phantom study (Fig. 4). The phantom (Fig. 4a) contained water, gelatin, and titanium dioxide (TiO_2_), in a weight ratio of 1000:100:1.5, respectively [45]. Three 90-µm thick black thread targets were placed in the phantom at a distance of 6 mm each in the lateral and axial directions starting from a depth of 12 mm from the phantom’s surface (Fig. 4b). Scans were performed along the Y-scan direction to quantify peak SNRs (pSNRs), and the axial and lateral resolutions of the systems in each target. In addition, scans along the X-scan direction were conducted to measure elevational resolutions of the systems in each target. Single-wavelength PA imaging was conducted at 797 nm, and thus the $y_{\mathrm{Scan}}$, $T_{\mathrm{Scan}}$, and FOVs for the operation of the updated scanner were 25 mm, 16.7 s, and 38.4 × 25 mm^2^, respectively. The previous scanner had an R of 20 mm, and thus different values of $y_{\mathrm{Scan}}$ and $T_{\mathrm{Scan}}$ were used to create the same step size (dy) as the updated scanner. Thus, the $y_{\mathrm{Scan}}$, $T_{\mathrm{Scan}}$, and FOV for the operation of the previously designed scanner were 33 mm, 22 s, and 38.4 × 33 mm^2^, respectively. To evaluate the repeatability of the systems, each scan was repeated three times at the same location. During each scanning, we have applied sufficient pressures on the scanner to minimize shaking and resulting artifacts. The PA imaging results from the phantom using the two scanners were shown in Fig. 4c-h. The locations of B-mode PA images shown in Fig. 4c and d correspond to the green dotted lines shown in Fig. 4e and f. The three black threads, labeled as #1-#3, were well visualized in all the cross-sectional PA and PA MAP images. The maximum PA imaging depth was 24 mm for thread #3. Although we carefully applied pressure on the scanner to minimize the artifacts, some image distortions were identified in Fig. 4g. We plan to minimize the moving artifacts by applying various motion correction algorithms using the similarity of successively scanned images in the near future. We also suspect there could be acoustic interference patterns in the lateral axis (i.e., along the thread), and advanced demodulation and envelope detection techniques could be investigated to solve the issue. To quantify the average pSNRs (ApSNRs) of the threads, each thread signal area was marked with pink boxes (Fig. 4c and d), and noise areas, bounded by gold boxes in Fig. 4c and d, were selected in each image frame in the three volume data set. The ApSNRs of the threads at different depths in the whole volume data set were plotted in Fig. 4i. Both the updated and the previous handheld scanners showed linearly decreasing ApSNRs (1.20 and 1.48 dB/mm, respectively) along the axial direction, corresponding to the optical attenuation of biological tissues [45]. The updated system achieved the ApSNRs of 59.8, 53.9, and 45.3 dB, respectively. At the same thread depths, the older system had ApSNRs of 51.2, 41.7, and 33.6 dB, respectively. Thus, the updated imaging system’s ApSNRs were better by 10.8 dB on average in the phantom. Notably, the water tank of the previous scanner used 0.44 mm thick PVC membranes to prevent leakage, but they could have created multiple layers of acoustic impedance mismatch. Further, the attenuation coefficient of the PVC was about 100 times higher than that of the US gel pad at the center frequency of the US transducer [46,47]. Thinner membranes could be an alternative, but they might tear and cause water leaking in clinical use.

The full width at half maximums (FWHMs) of the PA amplitudes of the threads in the axial, lateral, and elevation directions in each image frame were measured to quantify the average profiles of the threads in each direction in the whole volume data set. The newly updated handheld imaging system provided axial, lateral, and elevation profiles of 195 ± 3, 592 ± 20, and 1976 ± 53 μm, respectively, and the previously designed handheld imaging system provided axial, lateral, and elevation profiles of 237 ± 15, 646 ± 45, and 2386 ± 181 μm, respectively. The PA elevation profile results were wider than the US elevation beam-width. One of the reasons could be that US signals in the elevation direction were focused in both transmission and reception directions by a US lens, whereas PA signals were focused only during reception. Further, PA signals could be consisting of lower center frequencies than US center frequency of 8.5 MHz. In this study, we conservatively determined the scan step size using only the provided US round-trip beam-width of the US transducer.

### 3.2 Panoramic imaging of human arms and necks in vivo

The clinical feasibility of the 3D PA/US imaging system was verified through various 3D PA/US imaging experiments on the human arm and neck, following the protocols of the Institutional Review Boards of Yeungnam University Hospital (YUMC, IRB 2020–09–014) and Pohang University of Science and Technology (POSTECH, PIRB-2020-E019). All human subjects and examiners wore goggles for laser safety, and the maximum pulsed laser energy used was 10.7 mJ/cm^2^ [48] at 756 nm, less than the American National Standards Institute (ANSI) safety limit of 25.9 mJ/cm^2^.

**Fig. 5:**
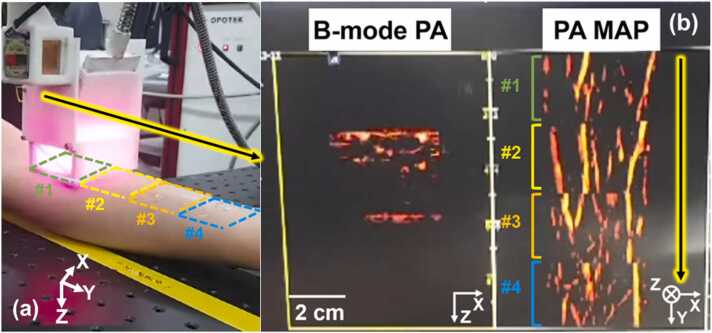
(a) Snapshot of online panoramic imaging experiments in vivo (Supplementary Video S1). #1-#4 and their dotted boxes represent each 3D PA/US imaging regions. (b) Snapshot of online B-mode PA and PA MAP images. Yellow-framed black arrows indicate the 3D scanning direction. PA, photoacoustic; and MAP, maximum amplitude projection.

To demonstrate online panoramic image display, a single-wavelength of 866 nm was used and four consecutive scans were performed on a volunteer’s forearm. The FOV was 38.4 × 100 mm^2^ and T_Scan_ was 66.8 s excluding 10 s of countdown before the laser emission. Fig. 5a shows a snapshot of the panoramic 3D arm imaging using the updated handheld scanner, with the scans labeled #1-#4 and corresponding colored and dashed rectangles outlining each imaged region. A yellow-framed black arrow indicates the panoramic imaging scan direction. Fig. 5b shows B-mode PA and PA MAP images displayed on the US imaging machine in real-time, and the PA MAP showed the super wide-field image of the human forearm vasculature. The FOV could be extended by increasing the number of scans, but there is a trade-off between n and $T_{\mathrm{Scan}}$. Previously in [25], because 3D images could not be immediately visualized during image acquisition, it was difficult to detect and discard motion-affected data. The updated system overcomes the problem by providing online preview of the PA MAP image, increasing its usability. Some discontinuities were identified in the Fig. 5b due to the manual positioning of the scanner for each 3D imaging. To overcome the limitation, we performed mosaic stitching using the 3D PHOVIS. The mosaic stitching and various post processing such as depth-encoding and volume rendering were available on the US machine right after panoramic 3D data acquisition was completed (**Supplementary Video S1**).

**Fig. 6:**
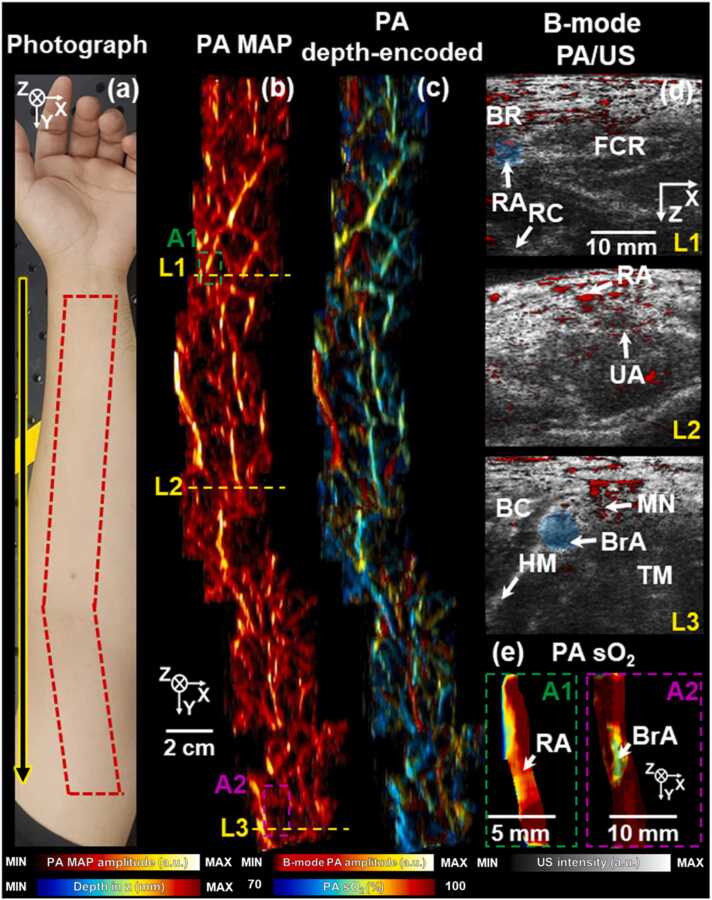
Panoramic imaging of a human arm. (a) A photograph of the human arm. A yellow-framed black arrow indicates the panoramic and scanning directions. A red dashed (and slightly crooked) rectangle indicates the imaged regions. (b) PA MAP and (c) PA depth encoded images. (d) B-mode PA Hb/US images corresponding to lines L1, L2, and L3, respectively. Blue semi-transparent polygons represent manually segmented regions. (e) PA sO_2_ images of manually segmented RA and BrA in regions A1 and A2, respectively. sO_2_, oxygen saturation; BR, brachioradialis muscle; RA, radial artery; RC, radial cortex; FCR, flexor carpi radialis; UA, ulnar artery; BC, biceps muscle; HM, humerus; BrA, brachial artery; TM, triceps muscle; MN, median nerve; PA, photoacoustic; MAP, maximum amplitude projection; and US, ultrasound.

To demonstrate offline panoramic 3D human arm imaging, we conducted 18 scans, resulting in the $T_{\mathrm{Scan}}$ of 901.8 s excluding the laser operation time, and the FOVof 331 × 38 mm^2^ along the elevation and lateral directions, respectively. In each 3D PA/US imaging, the scanner position was manually advanced along the elevation direction, and scanning was performed according in the scan direction (directions are represented by a yellow-framed black arrow in Fig. 6a). Multiple wavelengths of 756, 797, and 866 nm were used. In the photograph of the human arm, the imaged regions are indicated by a red dashed rectangle. The ROI between the elbow and the armpit was crooked to track the BrA. Fig. 6b and c show PA MAP and PA depth-encoded images, respectively. Volume-rendered results are shown in **Supplementary Video S2.** The distribution of blood vessels in different depth regions could be identified. The display resolutions of the PA MAP and PA depth encoded images were downgraded because the computational resources of the computer were insufficient to cover a large 3D panoramic data. Fig. 6d shows superimposed B-mode PA/US images correspond to the yellow dashed lines L1-L3 in Fig. 6b. The B-mode US image at L1 shows the brachioradialis muscle (BR), RA, flexor carpi radialis (FCR), and radial cortex (RC). The B-mode PA image at L1 clearly visualizes the RA’s boundaries. The RA and ulnar arteries (UA) meet near the elbow to form the BrA in the axillary direction, and the RA and UA were shown in the B-mode PA/US image at L2 before they merge into the BrA. The biceps muscle (BC), median nerve (MN), triceps muscle (TM), humerus (HM), and BrA could be identified in the B-mode US image at L3, and the B-mode PA image at L3 showed the BrA’s upper boundary. Due to compression by the scanner, the radial veins, ulnar veins, and brachial veins could not be visualized in all the B-mode PA/US images. Fig. 6e shows median-filtered PA sO_2_ MAP images of the RA and BrA at the ROI locations marked by A1 and A2, respectively. The size of the median filter was approximately 4 × 1 mm^2^ in the elevation and lateral directions, respectively. ROIs were manually segmented on all B-mode PA images, as indicated by the blue semi-transparent polygons in Fig. 6d. The mean ± standard deviation values of the average sO_2_ values for the RA and BrA are 95 ± 7.3 and 97 ± 6.9%, respectively, which are close to known values [34]. The structural and functional information provided by the images discussed above could be useful in diagnosing peripheral arterial disease (PAD) which can lead to serious complications, such as limb amputation and is coupled with an increased risk of cardiovascular events and death [49]. PAD can develop when the arteries supplying blood to the extremities become blocked, primarily due to atherosclerosis, impairing tissue perfusion and leading to hypoxia [50]. Complementing the conventional methods such as ankle-brachial index (ABI) test, PA imaging could provide information on sO_2_ and vessel blood perfusion when arm arteries are occluded, aiding in diagnosing PAD [51].

Supplementary material related to this article can be found online at doi:10.1016/j.pacs.2023.100512.

The following is the Supplementary material related to this article Video S2..

Video S2

**Fig. 7:**
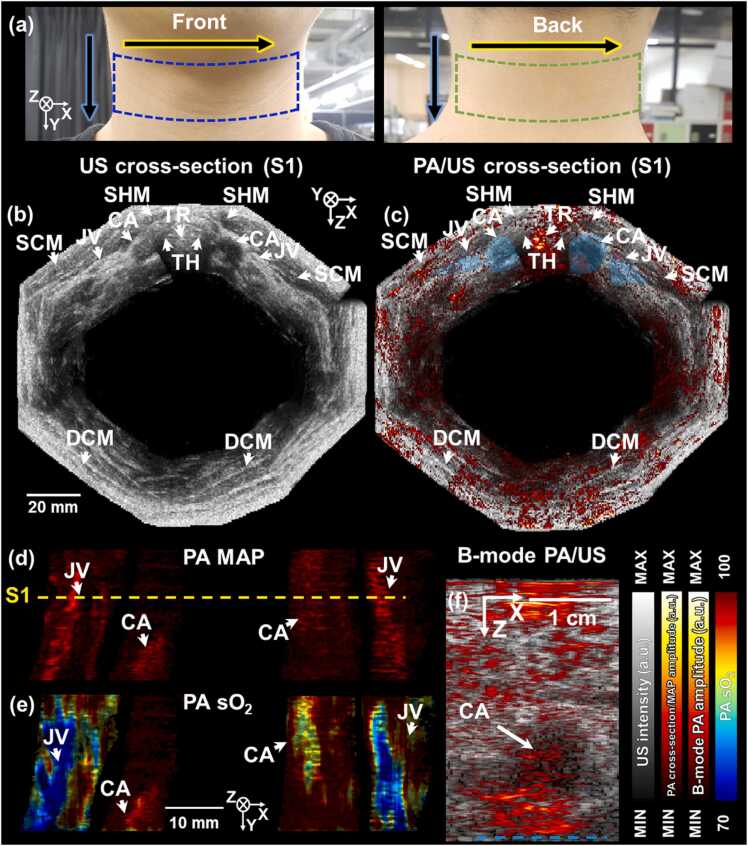
Panoramic 3D PA/US imaging of a human neck. (a) A photograph of the neck. Blue and green dashed polygons indicate imaged regions at the front and back of the neck, respectively. (b) US and (c) PA/US cross-section images of US and PA/US volume-rendered images, respectively. Blue semi-transparent polygons represent manually segmented regions of CAs and JVs. (d) PA MAP and (e) PA sO_2_ images of CAs and JVs, which are manually segmented. S1 corresponds to the cross-section position in (b) and (c). (f) B-mode PA/US images of human neck. A dotted jade line indicates the location of the maximum PA penetration depth, which is the lower boundary of CA. MAP, maximum amplitude projection; sO_2_, oxygen saturation; CA, carotid artery; JV, jugular vein; TH, thyroid; TR, trachea; SCM, sternocleidomastoid muscle; SHM, sternohyoid muscle; DCM, deep cervical muscles; US, ultrasound; and PA, photoacoustic.

We also demonstrated panoramic 3D imaging of the human neck using the same laser wavelength as for panoramic 3D imaging of the arm. We performed 12 scans around the neck to result in the $T_{\mathrm{Scan}}$ of 601.2 s excluding the laser operation time, and the transversal plane FOV of 129 × 120 mm^2^. Photographs of the volunteer’s neck are shown in Fig. 7a, where the imaged regions at the front and back of the neck are indicated by blue and green dashed polygons, respectively. The panoramic imaging direction and scanning direction are indicated by yellow and blue framed black arrows. Fig. 7b and c show US and PA/US cross-section images from US and PA/US volume-rendered images. The cross-section images were sectioned in the depth direction at the S1 position marked in Fig. 7d. Various morphological features such as CA, JV, thyroid (TH), trachea (TR), sternocleidomastoid muscles (SCM), sternohyoid muscle (SHM), and deep cervical muscles (DCM) could be identified in the US cross-section image, and the PA cross-section image visualized the CAs and JVs. The US and PA/US volume-rendered results can be seen in **Supplementary Video S3**. The neck surface appeared to be flat because the scanner pressed the neck to provide acoustic coupling without air gaps in the US gel. We expect that the surface could become smoother by increasing the number of acquired volumes around the neck. The PA contrast in the semi-transparent regions in Fig. 7c is relatively small because CAs and JVs were located at relatively deep depths. Using US transducers having wide aperture and broad bandwidth down to lower frequencies would be fundamentally helpful to achieve better contrast in deep biological tissues. Fig. 7d and e show PA MAP and median-filtered PA sO_2_ images of the segmented JVs and CAs, respectively. Median filtering was applied in the elevation and lateral directions with the same size as the arm imaging. The CA and JV were manually segmented in the same way as segmenting the ROIs in the arm. The ROIs in the cross-sectional area are indicated by blue semi-transparent polygons in Fig. 7c. The mean ± standard deviation values of the averaged sO_2_ values for the CAs and JVs were 97 ± 8.2 and 84 ± 3.8%, respectively, which are close to previously reported values [34,35]. To identify the maximum PA depth, we additionally imaged a volunteer’s neck with deep CA (Fig. 7f). We used a single wavelength of 866 nm and a single scan, and thus the FOVwas 38.4 × 25 mm^2^ and $T_{\mathrm{scan}}$ was 66.8 s. The maximum PA penetration depth at the lower boundary of the CA indicated by the dotted jade line was 28.6 mm, an increase of 13.9 mm compared to the previous system. A potential usage of this panoramic neck imaging is detecting the CA stenosis which begins with atherosclerosis of the CA and eventually leads to a hypovolemic cerebral infarction or an embolic stroke. About 23% of ischemic strokes result from rupture of carotid plaques [52], and the rate of reduction in arterial diameter due to these plaques is used as a measure of stroke risk. Currently, the degree of stenosis of the CA and plaque are mainly non-invasively measured using 2D duplex US imaging, but it is impossible to accurately quantify the plaque and determine its characteristics. We expect that accurate quantification of atherosclerotic plaques via morphological and multispectral analysis of 3D PAUS imaging will be helpful in diagnosis.

Supplementary material related to this article can be found online at doi:10.1016/j.pacs.2023.100512.

The following is the Supplementary material related to this article Video S3..

Video S3

## 4 Conclusion

In this study, we demonstrated a super-wide field human vasculature imaging by using the panoramic 3D handheld PA/US imaging. We updated 3D PHOVIS to provide mosaic stitching in 6 degrees-of-freedom, which made it applicable for coregistering multiple volume images of curved structures (e.g., human neck). To test the feasibility of the developed imaging system, we tested 3D panoramic imaging on the human arm and neck. Maximum FOV for the arm and neck were 331 × 38 and 129 × 120 mm^2^, respectively, and the corresponding scanning times were 901.8 and 601.2 s, respectively. The reliability of the system was confirmed through sO_2_ quantification of major blood vessels. Based on these results, the newly updated system is expected to be potentially useful in diagnosing cardiovascular, vascular surgery, internal medicine, skin diseases and cancers. In the future, we plan to develop automatic position correction for 3D panoramic imaging, which is expected to greatly improve the clinical usefulness of the PA/US imaging system. Currently, it is difficult to find feature points due to various factors such as US speckle and PA noise, making the automatic correction challenging. We also plan to develop online ROI quantification for the analysis of various clinical indicators (e.g., PA amplitude, PA hemoglobin, PA vascular density, PA melanin, PA lipids, and PA sO_2_) implemented with real-time laser power calibration. Further, we plan to reduce scan time by using a laser system that provide a faster PRF while maintaining the laser power, using more US receiving channels, and by optimizing scan step size.

## Declaration of Competing Interest

C. Lee, S. Cho, D. Lee, J. Lee, J. I. Park, H. J. Kim, S. H. Park, W. Choi, and U. Kim declare no competing interests. C. Kim has financial interests in OPTICHO, which, however, did not support this work.

## Data Availability

Data will be made available on request.
