# Supplementary material for: Panoramic volumetric clinical handheld photoacoustic and ultrasound imaging
Source: Photoacoustics. 2023 May 18;31:100512. doi: 10.1016/j.pacs.2023.100512 (PMC10208888; doi:10.1016/j.pacs.2023.100512)
Supplement: Supplementary file 4 — Supplementary material [file mmc1.docx]

**Supplementary information for:**

**Manuscript number: PACS-D-23-00052**

**Manuscript title: Panoramic Volumetric Clinical Handheld Photoacoustic and Ultrasound Imaging**

**Authors and affiliations**

Changyeop Lee^1^, Seonghee Cho^1^, Donghyun Lee^1^, Jonghun Lee^1^, Jong-Il Park^2^,
Hong-Ju Kim^3^, Sae Hyun Park^4^, Wonseok Choi^5,*^, Ung Kim^2,*^, and Chulhong Kim^1,*^

^1^Departments of Electrical Engineering, Convergence IT Engineering, Mechanical Engineering, and Medical Science and Engineering, Medical Device Innovation Center, Pohang University of Science and Technology, Pohang 37673, Republic of Korea.

^2^Division of Cardiology, Department of Internal Medicine, Yeungnam University Medical Center, Yeungnam University College of Medicine, Daegu 42415, Republic of Korea.

^3^Division of Cardiology, Department of Internal Medicine, Severance Hospital, Seoul 03722, Republic of Korea.

^4^Division of Cardiology, Department of Internal Medicine, Daegu Veterans Hospital, Daegu 42835, Republic of Korea.

^5^Department of Biomedical Engineering, College of Medicine, The Catholic University of Korea, Seoul 06591, Republic of Korea.

*Corresponding authors:
Chulhong Kim ([chulhong@postech.edu](mailto:chulhong@postech.edu))
Ung Kim ([woongwa@yu.ac.kr](mailto:woongwa@yu.ac.kr))
Wonseok Choi ([wonseok.choi@catholic.ac.kr](mailto:wonseok.choi@catholic.ac.kr))

**Table of contents**

**Supplementary Figures**

Figure. S1. Schematic description of the scotch yoke mechanism 1

Figure. S2. Data acquisition scheme for a single PA image frame 2

**
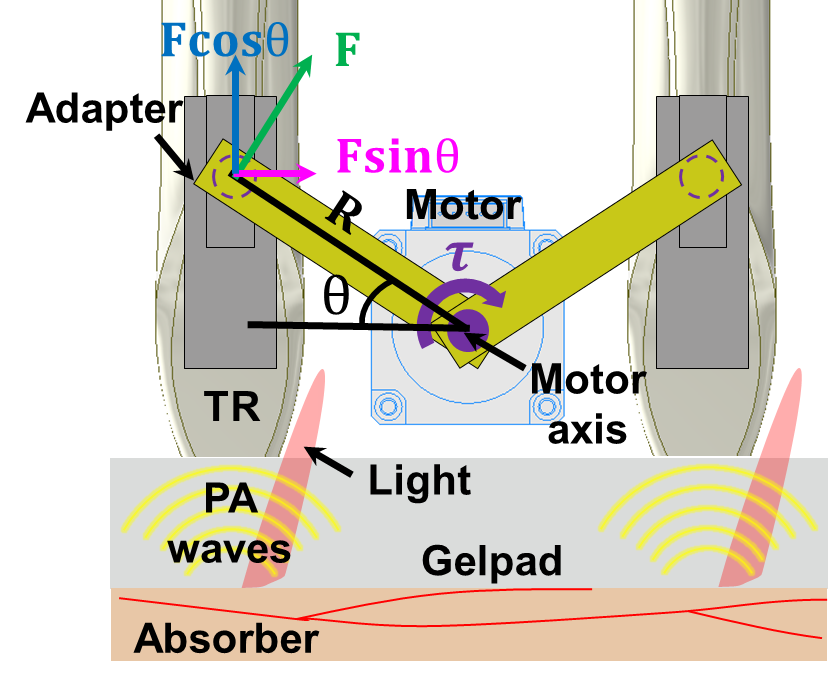
**

**Supplementary Figure S1.** Schematic description of the scotch yoke mechanism. When motor begins to rotate the arm, torque $\tau$ and resultant force F (=$\tau\div R$) is generated at the tip of the arm. The vertical force Fcosθ does not affect the adapter motion because the arm tip moves freely along the groove. The horizontal force Fsinθ moves the adapter linearly in the scanning direction. PA, photoacoustic; and TR, ultrasound transducer.


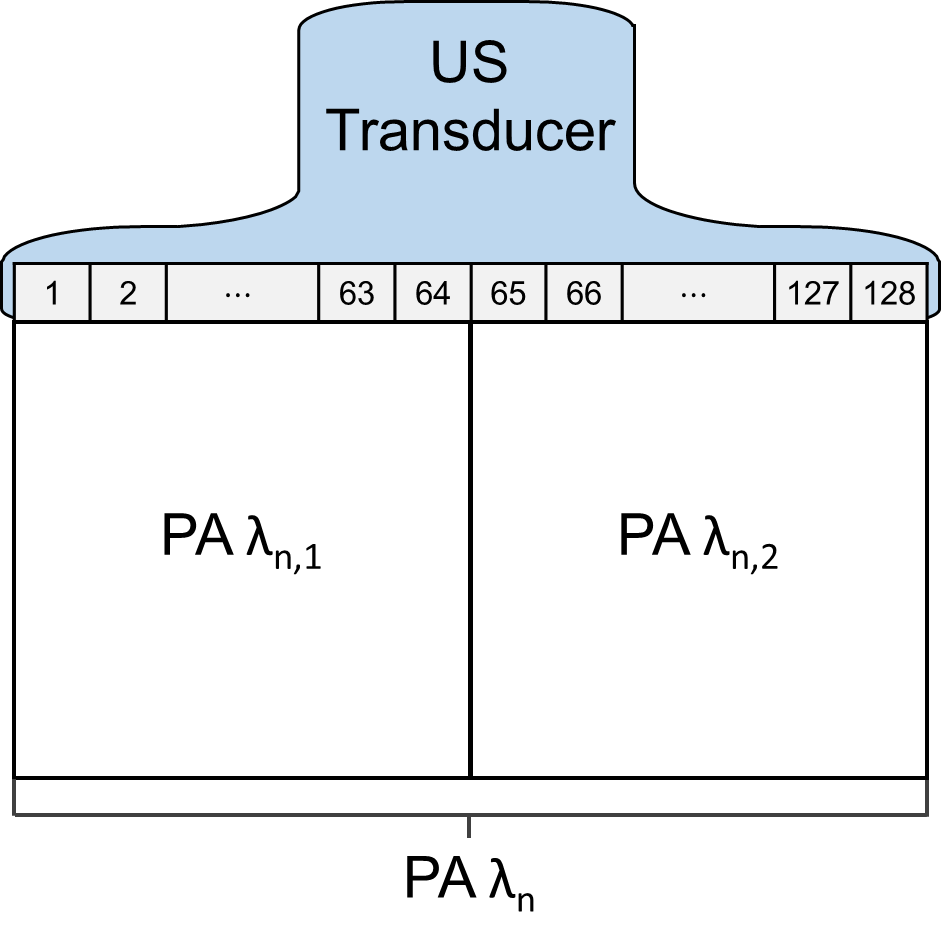


**Supplementary Figure S2.** Data acquisition scheme for a single PA image frame. Two laser pulses were used to form a single PA frame of a 128-element US transducer because the US system had 64 data acquisition channels. The first laser shot λ_1,1_ induced PA data for elements #1-#64, and the second laser shot λ_1,2_ for elements #65-#128. PA, photoacoustic; US, ultrasound; and TR, transducer. n represent the number of laser wavelength.
